# The prognostic significance of lactate dehydrogenase levels in seminoma patients with advanced disease: an analysis by the Global Germ Cell Tumor Collaborative Group (G3)

**DOI:** 10.1007/s00345-021-03635-3

**Published:** 2021-03-08

**Authors:** Christoph Seidel, Gedske Daugaard, Tim Nestler, Alexey Tryakin, Mikhail Fedyanin, Christian Daniel Fankhauser, Thomas Hermanns, Jorge Aparicio, Julia Heinzelbecker, Pia Paffenholz, Axel Heidenreich, Ugo De Giorgi, Richard Cathomas, Anja Lorch, Anna Fingerhut, Fabian Gayer, Felix Bremmer, Patrizia Giannatempo, Andrea Necchi, Daniele Raggi, Gaetano Aurilio, Chiara Casadei, Marcus Hentrich, Ben Tran, Klaus-Peter Dieckmann, Margarido Brito, Christian Ruf, Alessandro Mazzocca, Bruno Vincenzi, Olof Stahl, Carsten Bokemeyer, Christoph Oing

**Affiliations:** 1 Department of Oncology, Hematology and Bone Marrow Transplantation with Division of Pneumology, University Medical Center Hamburg-Eppendorf, Martinistraße 52, 20246 Hamburg, Germany; 2 Department of Oncology, Copenhagen University Hospital, Rigshospitalet, Copenhagen, Denmark; 3 Department of Urology, Federal Armed Services Hospital Koblenz, Koblenz, Germany; 4 Department of Clinical Pharmacology and Chemotherapy, N. N. Blokhin Russian Cancer Research Center, Moscow, Russian Federation; 5 University Hospital Zürich, Zurich, Switzerland; 6 Medical Oncology Department, Hospital La Fe - On behalf of the Spanish Germ Cell Cancer Group, Valencia, Spain; 7 Department of Urology, University Hospital Saarland, Homburg/Saar, Germany; 8 Department of Urology, University Hospital Cologne, Cologne, Germany; 9 Department of Medical Oncology, Istituto Scientifico Romagnolo per lo Studio e la Cura Dei Tumori (IRST) IRCCS - On behalf of the Italian Germ Cell Cancer Group (IGG), Meldola, Italy; 10 Department of Oncology/Hematology, Kantonsspital Graubünden, Chur, Switzerland; 11 Department of Oncology and Hematology, University Hospital Zürich, Zurich, Switzerland; 12 Department of Urology, University Hospital Düsseldorf, Düsseldorf, Germany; 13 Department of Urology, University Clinic Göttingen, Göttingen, Germany; 14 Department of Pathology, University Clinic Göttingen, Göttingen, Germany; 15 Fondazione IRCCS, Istituto Nazionale dei Tumori, Milan, Italy; 16 Medical Division of Urogenital and Head and Neck Cancer, IEO European Institute of Oncology IRCCS, Milan, Italy; 17 Department of Hematology and Oncology, Red Cross Hospital, University of Munich, Munich, Germany; 18 Peter MacCallum Cancer Centre, Melbourne, Australia; 19 Asklepios Klinik Altona, Hodentumorzentrum, Hamburg, Germany; 20 Instituto Português de Oncologia de Lisboa, Lisboa, Portugal; 21 University Campus Bio-Medico, Rome, Italy; 22 SWENOTECA, Trondheim, Norway; 23 Department of Oncology, Skane University Hospital, Lund, Sweden; 24 Mildred Scheel Career Center HaTriCS4, University Medical Center Eppendorf, Hamburg, Germany; 25 Department of Urology, Medical University Vienna, Vienna, Austria; 26 Walter and Eliza Hall Institute of Medical Research, Melbourne, Australia; 27 Department of Urology, Uro-Onology, Robot-Assisted and Specialized Urologic Surgery, University of Cologne, Cologne, Germany

**Keywords:** Seminoma, Lactate dehydrogenase, Prognostic markers, Upper limit of normal

## Abstract

**Purpose:**

The prognostic significance of lactate dehydrogenase (LDH) in patients with metastatic seminoma is not defined. We investigated the prognostic impact of LDH levels prior to first-line systemic treatment and other clinical characteristics in this subset of patients.

**Methods:**

Files from two registry studies and one single-institution database were analyzed retrospectively. Uni- and multivariate analyses were conducted to identify patient characteristics associated with recurrence free survival (RFS), overall survival (OS), and complete response rate (CRR).

**Results:**

The dataset included 351 metastatic seminoma patients with a median follow-up of 5.36 years. Five-year RFS, OS and CRR were 82%, 89% and 52%, respectively. Explorative analysis revealed a cut-off LDH level of < 2.5 upper limit of normal (ULN) (*n* = 228) vs. ≥ 2.5 ULN (*n* = 123) to be associated with a significant difference concerning OS associated with 5-years OS rates of 93% vs. 83% (*p* = 0.001) which was confirmed in multivariate analysis (HR 2.87; *p* = 0.004). Furthermore, the cut-off LDH < 2.5 ULN vs. ≥ 2.5 ULN correlated with RFS and CRR associated with a 5-years RFS rate and CRR of 76% vs. 86% (*p* = 0.012) and 32% vs. 59% (*p*  ≤  0.001), respectively.

**Conclusions:**

LDH levels correlate with treatment response and survival in metastatic seminoma patients and should be considered for their prognostic stratification.

## Introduction

Germ cell tumors (GCTs) are the most common solid malignancy in young men aged between 15 and 35 years [1]. Metastatic GCTs with both seminomatous and non-seminomatous histology are extremely sensitive to cisplatin-based chemotherapy as part of a multimodal treatment approach associated with 5-years overall survival (OS) rates from 50% to 90% depending on the risk group [2, 3]. The serum tumor markers beta subunit of human chorionic gonadotropin (HCG) and alpha-fetoprotein (AFP) are used for diagnosis, monitoring of treatment efficacy, and follow-up procedures [4]. In patients with non-seminomatous GCTs, the serum tumor markers HCG, AFP and LDH (lactate dehydrogenase) are used for the prognostic stratification according to the International Germ Cell Cancer Cooperation Group (IGCCCG) classification; however, tumor markers are not considered for further prognostic estimations of seminoma patients. Only approximately 30% of seminoma patients with advanced disease have serum HCG levels above normal [5–7], and unequivocally increased AFP levels are not consistent with pure seminomatous histology. In the previous work of our group, we analyzed a cohort of seminoma patients with elevated HCG levels and demonstrated that HCG levels are not associated with the outcome except in a small subset of advanced-stage patients with excessive HCG marker increases detected pre-orchiectomy [8]. Although pre-orchiectomy tumor markers are principally not considered for the prognostic stratification of GCT patients [9, 10] the results of our study revealed that HCG is not sufficient as a prognostic marker for seminoma patients [11]. Already in 1981, elevated LDH serum levels were reported to correlate with tumor bulk and impaired survival times in a small series of GCT patients [12], while other studies revealed a correlation between LDH serum levels and disease burden in GCT patients [7, 13]. The prognostic significance of LDH levels in patients with pure seminomatous histology, however, was never yet defined. This study examines the prognostic impact of LDH levels detected prior to first-line systemic treatment concerning recurrence-free survival (RFS), overall survival (OS), and complete response rate (CRR) after forming an extended group of seminoma patients with advanced disease.

## Patients and methods

### Study population and inclusion criteria

We analyzed a contemporary cohort of seminoma patients who had been treated from 1983 to 2017. The patients were retrospectively identified in 20 GCT expert centers or study groups across Europe, the Russian Federation, and Australia that participated in two previous registry studies on behalf of the global germ cell collaborative group (G3) with additional data from one single institution analysis. Previous registry studies provided data for the analysis of intermediate prognosis patients in metastatic GCT and for HCG positive seminoma patients, respectively [8, 14]. Registry studies were approved by the Ethics Committee of the Medical Association, Hamburg, Germany (Ref. No.: PV5432 and PV5050). Monocentric data acquisition was in line with local requirements according to Hamburg Hospital Act (HmbKHG)§ 12 HmbKHG. Patients were eligible for inclusion if they had (1) confirmed pure seminomatous histology according to histological examination by local pathologists, (2) advanced disease including the clinical stages IIA-C and IIIA-C according to Union for International Cancer Control (UICC) [15], (3) systemic treatment (4) availability of LDH levels detected prior to first-line systemic treatment (i.e. post-orchiectomy if carried out). Patients were excluded due to the following reasons: (1) non-seminomatous histology, (2) localized disease or (3) absence of LDH levels prior to first line treatment (4) treatment with radiotherapy only. LDH levels prior to first-line systemic treatment were determined in institutional laboratories according to international practice guidelines [16]. Due to variable measurement techniques applied in the participating institution with differences concerning reference ranges, we chose to analyze individual measurements as x-fold values of the upper limit of normal (ULN) according to local laboratory measurement technologies.

### Outcome measurements and endpoint

Our study hypothesis was that elevated LDH levels detected prior to first-line tretament inversely correlate with outcome in seminoma patients with metastatic disease. End-points of the study were, RFS defined as the time from the end of first line treatment to the date of disease recurrence, OS defined as the time from the first diagnosis until death from any cause or last date of follow-up, and complete response rate (CRR) defined as the rate of complete remission achieved during first-line chemotherapy. Complete remission was defined as no radiological evidence of residual disease and normalized tumor markers after completion of systemic treatment or radiotherapy. Patients lost to follow-up were censored at the date of their last visit.

### Statistical analysis

To explore a correlation between rising LDH levels and outcome patients were stratified according to the extent of their LDH levels detected prior to first-line treatment using the following model: (1) < 1.5 ULN vs. (2) 1.5 ULN to 2 ULN (3) vs. > 2–3 ULN, (4) vs. > 3.0 ULN. To find a cut-off level which reveals the highest difference between beneficial and impaired outcomes, the following cut-off levels were tested: LDH prior to first-line treatment ≥ 1.5 ULN vs. < 1.5 ULN; LDH prior to first-line treatment ≥ 2.0 ULN vs. < 2.0 ULN; LDH prior to first-line treatment ≥ 2.5 ULN vs. < 2.5 ULN; LDH prior to first-line treatment ≥ 3.0 ULN vs. < 3.0 ULN. Other potential prognostic indicators considered for statistical analysis were age, IGCCCG risk category, primary testicular vs. extragonadal, pulmonary vs. other visceral metastases, and HCG levels prior to first-line treatment (≥ 2.000 IU/l vs. < 2.000 IU/l and ≥ 5.000 IU/l vs. < 5.000 IU/l). Survival analysis was conducted using the Kaplan–Meier method [17]. A log-rank test was applied to compare survival rates. Patient characteristics found to be associated with outcome (*p* < 0.1) were tested in a multivariate Cox regression model. Variables were found to be significant if a two-sided *p* value was < 0.05. To analyze the correlation between patient characteristics and CRR Pearson Chi-square test was conducted. Statistical analysis was performed using Statistical Package for the Social Sciences (SPSS, version 17.0; IBM Corp, Armonk, NY, USA).

## Results

### Patient characteristics

**Fig. 1:**
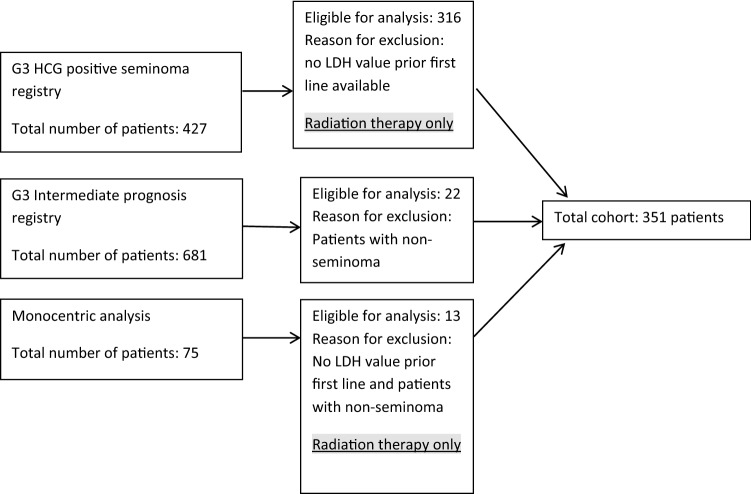
Overview of the patient cohort: total cohort divided into a number of patients in different analyses

Data files from two registry studies and one single institution database were screened. Of 1183 files 351 seminoma patients with metastatic disease were considered eligible for analysis (Fig. 1). Clinical characteristics of the patient cohort are described in Table 1. The median follow-up time since diagnosis was 5.36 years (interquartile range: 6.32). The cohort included patients with clinical stages IIA-C (*n* = 209) and IIIA-C (*n* = 129) according to UICC [14]. In 13 cases the exact stage of advanced disease remained undefined. LDH levels upper limit of normal prior to first-line treatment according to local laboratory findings were detected in 261 of 351 (74%).

**Table 1:** Patient characteristics

Characteristics	Absolute number of patients *n* = 351	100%
LDH values prior to first-line treatment (ULN)		
< 1.5	155	44%
> 1.5–2	54	15%
> 2–3	35	10%
> 3	107	30%

Median age in years	39.9	(Range 18–79)
Primary site of the tumor		
Gonadal	263	75%
Extragonadal	70	20%
Missing	18	5%
IGCCCG classification		
Good prognosis	276	78%
Intermediate prognosis	65	19%
Missing	10	3%
Stage UICC		
UICC Stage II	209	59%
UICC Stage III	129	37%
Missing	13	4%
Treatment regimens		
Three cycles of BEP	126	36%
Four cycles of BEP	122	35%
Three to four cycles VIP	8	2%
Four cycles of PE	18	5%
Four cycles of CE	4	1%
Defined as “others”	60	17%
Missing	13	4%

*UICC* Union for International Cancer Control, *BEP* Bleomycin, Etoposide, Cisplatin, *VIP* Vindensine, Ifosfamide, Cisplatin, *PE* Cisplatin Etoposide, *SAKK* Schweizerische Arbeitsgemeinschaft für Klinische Krebsforschung

### Treatment and response

Systemic cisplatin-based chemotherapy according to institutional standards was administered in 274 patients (78%). Altogether 60 systemic chemotherapy regimens were defined as ‘others’ (17%) while in 13 cases further information on treatment regimens was absent (4%). Additional surgery after systemic treatment was documented in 21 patients (6%). Treatment response data were available in 274 patients. Complete remissions were achieved in 145 (53%), partial remissions in 108 (39%), stable disease in 11 (4%), and progressive disease in 10 (4%) patients. Details of the treatment regimens are summarized in Table 1.

### Correlation of patient characteristics with overall survival (OS) and recurrence-free survival (RFS)

**Fig. 2:**
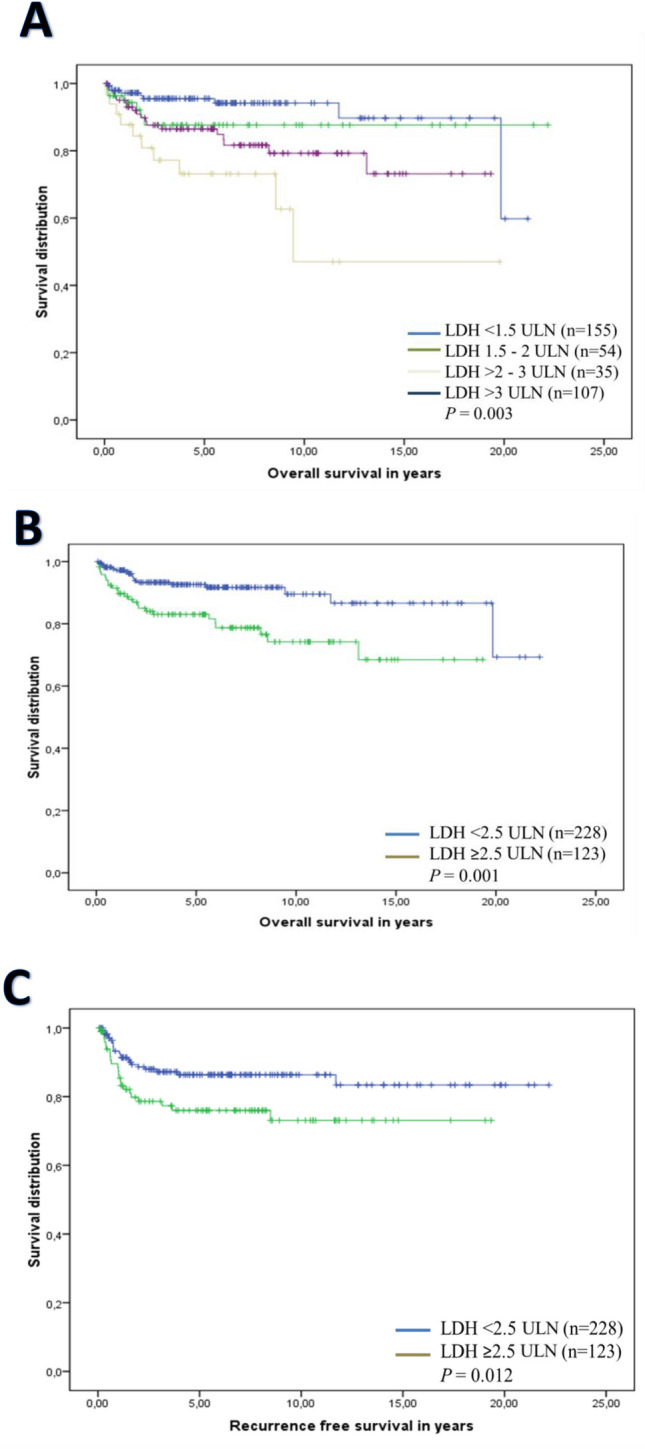
Patients were stratified into four cohorts according to increasing LDH levels detected prior to first-line treatment:  <  1.5 upper limit of normal (ULN); 1.5–2; from >  2 to 3, and >  3 ULN. The graph demonstrates a correlation between rising LDH levels and impaired outcome concerning OS (**a**). Stratification of patients according to prior to first-line treatment LDH levels identifies ≥ 2.5 ULN as a cut-off level with significant prognostic impact concerning OS (**b**) and RFS (**c**)

The 5-year OS rate of the total cohort was 89%. The stratification of patients according to their LDH levels detected prior to first-line treatment suggests an inverse correlation between rising LDH levels and outcome. Five year OS rates of 94% were detected for patients with LDH levels < 1.5 ULN (*n* = 155); 87% for patients with LDH levels from 1.5 ULN to 2 ULN (*n* = 54), 76% for patients with LDH levels from > 2 to 3 ULN (*n* = 35) and 86% for patients with LDH levels > 3.0 ULN (*n* = 107) (*p* = 0.003) (Fig. 2). To evaluate a cut-off providing the highest discrepancy between patients with good and impaired outcomes, patients were consecutively categorized according to their extent of LDH levels. Cut-off levels tested are described in “*Outcome measurements and endpoint*”. Regarding this stratification, our analysis revealed the cut-off level of LDH ≥ 2.5 ULN prior to first-line treatment (*n* = 123) vs. < 2.5 ULN (*n* = 228) providing the highest difference concerning outcome associated with a 5-year OS rate of 83% vs. 93% (*p* = 0.001) (Table 2; Fig. 2). Other factors correlating with an impaired OS rate were HCG levels ≥ 2.000 IU/l vs. < 2.000 IU/l (*n* = 12) detected prior to first-line treatment associated with a 5-year OS rate of 82% vs. 89% (*p* = 0.022) and age above the median revealed an impaired 5-year survival rate of 86% vs. 91% vs. (*p* = 0.024). Multivariate analysis confirmed LDH ≥ 2.5 ULN as the only independent prognostic factor concerning OS (HR 2.86; 95% CI 1.41–5.85; *p* = 0.004). Results of the outcome analysis are illustrated in detail in Table 2. Concerning the RFS, LDH cut-off levels and other patient characteristics were tested as well. The 5-year RFS rate was 83%. Univariate analysis revealed LDH levels prior to first-line treatment as the only variable to be associated with the RFS. Here LDH levels ≥ 2.5 ULN vs. < 2.5 ULN were associated with a five-year RFS rate of 76% vs. 86% (*p* = 0.012) (Table 2; Fig. 2). As no other variable was detected to be significant, further multivariate analysis was not conducted.

**Table 2:** Results of univariate and multivariate analyses of OS, RFS and CRR

Factor	Difference 5-year OS rate	Log-rank test *p* value
LDH prior to first-line < 1.5 ULN vs. ≥ 1.5 ULN	85% vs. 94%	0.021
LDH prior to first-line ≥ 2.5 ULN vs. < 2.0 ULN	84% vs. 94%	0.003
LDH prior to first-line ≥ 2.5 ULN vs. < 2.5 ULN	83% vs. 93%	0.001
LDH prior to first-line ≥ 3.0 ULN vs. < 3.0 ULN	87% vs. 91%	0.054
Age above vs. below median	86% vs. 91%	0.024
IGCCCG good vs. intermediate	88% vs. 90%	0.240
HCG prior to first line > 2000 U/l	82% vs. 89%	0.022
HCG prior to first line > 5000 U/l	87% vs. 89%	0.063

Factor	HR	95% CI	*p* value
LDH prior to first-line ≥ 2.5 ULN vs. < 2.5 ULN	2.87	1.41–5.85	0.004
HCG prior to first-line > 2000 U/l	0.41	0.14–1.19	0.102
Age above vs. below median	1.67	0.86–3.25	0.132

Factor	Difference 5-year RFS rate	Log-rank test *p* value
LDH prior to first-line ≥ 1.5 ULN vs. < 1.5 ULN	79% vs. 89%	0.027
LDH prior to first-line ≥ 2.0 ULN vs. < 2.0 ULN	78% vs. 86%	0.082
LDH prior to first-line ≥ 2.5 ULN vs. < 2.5 ULN	76% vs. 86%	0.012
LDH prior to first-line ≥ 3.0 ULN vs. < 3.0 ULN	78% vs. 86%	0.087
Age above vs. below median	81% vs. 83%	0.673
IGCCCG good vs. intermediate	82% vs. 84%	0.933
HCG prior to first-line > 2000 U/l	73% vs. 84%	0.552
HCG prior to first-line > 5000 U/l	72% vs. 82%	0.063

Factor	Difference CRR	Chi-square test *p* value
LDH prior to first-line ≥ 1.5 ULN vs. < 1.5 ULN	39% vs. 64%	0.005
LDH prior to first-line ≥ 2.0 ULN vs. < 2.0 ULN	48% vs.50%	< 0.001
LDH prior to first-line ≥ 2.5 ULN vs. < 2.5 ULN	32% vs. 59%	< 0.001
LDH prior to first-line ≥ 3.0 ULN vs. < 3.0 ULN	21% vs. 78%	< 0.001
Age ≥ median vs. < median	49% vs. 52%	0.655
Gonadal vs. extragonadal	78% vs.22%	0.480
IGCCCG good vs. intermediate	46% vs. 54%	0.382
HCG prior to first-line < vs. > 2000 U/l	53% vs. 57%	0.821
HCG prior to first-line < vs. > 5000 U/l	52% vs. 80%	0.221

*LDH* lactate dehydrogenase, *IGCCCG* International Germ Cell Cancer Collaborative Group

### Correlation of patient characteristics with complete remission rate

The complete remission rate was 53%. Chi-square test revealed LDH levels prior to first-line treatment to inversely correlate with CRR. Here LDH levels ≥ 2.5 ULN vs. < 2.5 ULN were associated with a CRR of 32% compared to 59% (*p* < 0.001), respectively. None of the other clinical characteristics significantly correlated with CRR (Table 2).

## Discussion

This registry study proved a significant correlation of serum LDH levels with OS, RFS and CRR in patients with metastasized seminoma. Elevated LDH levels were already associated with poorer outcomes including several solid tumor and hematologic malignancies [18–22] but the value of LDH as a prognostic marker for seminoma patients remained unclear. To find new categories that significantly correlate with outcome, our analysis investigates LDH levels and other patient characteristics as potential prognostic markers, hypothesizing that LDH inversely correlates with outcome in seminoma patients as well. We herein found a negative correlation between rising LDH levels and outcome concerning OS in consideration of uni- and multivariate analyses. According to different LDH thresholds, the cut-off level of ≥ 2.5 ULN vs. < 2.5 ULN provided a high discrepancy between patients with impaired and beneficial outcomes. Of note, this cut-off was also significantly relevant concerning RFS and CRR within our analysis and is in line with results recently reported by the EORTC IGCCCG-update consortium [23].

This analysis can add substantially new information to the field of testis cancer by identifying LDH levels > 2.5 ULN as a novel marker to define seminoma patients at high risk.

Limitations of the study are a retrospective design and partially incomplete datasets. Another limitation is that the majority of patients were identified from an earlier registry study including exclusively seminoma patients with HCG levels above normal reference ranges. As our previous study demonstrated that HCG levels are not associated with the outcome except in a small subset of advanced-stage patients with excessive HCG marker increases detected pre-orchiectomy we think that this large subset of marker positive HCG levels in our cohort will not interfere with data of seminoma patients without any elevated HCG levels. We therefore consider the value of elevated LDH levels to identify seminoma patients with an inferior prognosis as promising new information, representing an easily applicable assessment for the daily clinical routine. Here strategies for treatment escalation or de-escalation based on LDH might be worth to be considered for future projects.

## Conclusions

This retrospective series of advanced seminoma patients highlights the prognostic impact of LDH levels detected prior to first-line systemic treatment concerning OS, RFS and CRR.

LDH levels expound a distinct utility to characterize a subgroup of seminoma patients with an inferior outcome.

Additional research to further evaluate the potential utility of this marker for prognostic stratification and treatment adjustments is needed.
